# Validity Prediction of Amplitude-Integrated EEG in Early Neuromotor Development Outcomes in High-Risk Neonates

**DOI:** 10.1155/2020/9438248

**Published:** 2020-03-11

**Authors:** Jian Guo, Wentao Wang, Qili Zhou

**Affiliations:** Affiliated Hospital of Chengde Medical College, Chengde, Hebei 067000, China

## Abstract

With the continuous advancement of medical technology, the survival rate of high-risk children is increasing year by year, but the developmental problems that have gradually become apparent in the later stages have a serious impact on the quality of life of children. Amplitude-integrated EEG is an EEG monitoring technology developed for clinical use in newborns in recent years. Therefore, to better detect neuromata development in high-risk children, this study explores the validity prediction of amplitude-integrated EEG in early neuromata development in high-risk children. For 100 high-risk children, amplitude-integrated EEG was used for monitoring, and the exercise scale and validity predictors in the Bailey Infant Development Scale were used to assess whether high-risk children had neurobehavioral abnormalities. The experimental results show that the application of amplitude-integrated EEG can make accurate and effective predictions of early neuromata development outcomes in high-risk children. Compared with traditional neurological examination methods, it has higher sensitivity, specificity, positive predictive value, and consistency in predicting the early neuromata development outcomes of high-risk children. It is suitable for application and promotion in China and has a good application value.

## 1. Introduction

With the continuous advancement of medical technology, the survival rate of high-risk children is increasing year by year. However, the developmental problems that have gradually become apparent in the later period have severely affected the quality of life of children, including audio-visual impairment, cerebral palsy, and mental retardation [1]. In recent years, the incidence of high-risk children has continued to rise and has now reached about 8–10% [2]. And with the development of the technology of neonatal intensive care unit (NICU), the survival rate of high-risk children is getting higher and higher, especially the survival rate of very low birth weight children and ultralow birth weight children is significantly improved. Along with this, increased attention has been paid to the issue of nerve injury in high-risk children. Studies have found that almost 25% of high-risk children have potential neurodevelopmental problems [3]. Therefore, we should pay attention to the early detection, correct diagnosis, and early intervention of nerve injury in high-risk children.

Objective diagnosis and evaluation methods of nerve injury and nerve development mainly rely on imaging methods such as craniocerebral ultrasound and MRI. Due to technical limitations, early dynamic monitoring is difficult. In recent years, more and more studies have found that [4, 5] although the electroencephalogram (EEG) is not specific for the diagnosis of nerve damage, it is one of the sensitive indicators for judging the function of the brain. Damage usually precedes damage to the nerve structure. Therefore, EEG can be used as an objective evaluation index of early nerve injury and prognosis. However, due to problems such as electroencephalography and analysis techniques, clinical practice has not been widely available. At present, there are many methods to detect neuromotor development, but the standards of various methods are different. It is important to screen out an effective method for assessing the neuromotor development outcomes of high-risk children. Amplitude-integrated electroencephalogram (aEEG) and general movement (GM) quality assessment is a sensitive assessment system for neural injury that has emerged in recent years, especially for the assessment of neurodevelopmental outcomes in newborns and infants [6]. Technology aEEG is an EEG analysis method, which is mainly analysed by amplitude waves. It has good consistency with conventional EEG, but its advantages in background waveforms or classification are more significant. It can not only be bedside but also long-term, continuous monitoring of brain function makes it easier to judge brain function [7]. GM is a simple and noninvasive operation. Its quality changes when the nervous system is damaged, and various abnormal features appear. It has been proven that GM quality assessment technology has a good early predictive value for cerebral palsy [8]. However, the quality assessment of GMs has limited ability to predict mild neurological dysfunction, and the prediction of neurodevelopmental outcomes in high-risk children is not as good as aEEG [9]. Compared with EEG and GMs, on the one hand, because there are fewer aEEG electrodes, it is convenient for long-term recording, and it is especially suitable for bedside brain function monitoring of high-risk children in NICU. On the other hand, aEEG is easy to operate, intuitive in graphics, and easy to analyse. Many studies have shown that good consistency can be maintained between aEEG and conventional EEG [10, 11]. Goswami et al. [12] showed that according to the acquisition and classification of background waveforms, aEEG has advantages over conventional EEG. Filippi et al. [13] demonstrated in their experiments that they did not find any complications caused by aEEG recording or the use of needle electrodes, indicating that long-term aEEG recording is safe and feasible.

aEEG is a neonatal brain function monitoring device newly introduced in clinical practice in recent years, which can realize continuous real-time monitoring at the bedside, can reflect changes in EEG background activities and epilepsy-like activities, and is very sensitive to newborn brain development and can be diagnosed as early brain injury has become one of the routine monitoring items in the clinical neonatal care unit [14]. Technology aEEG abnormalities suggest a poor prognosis for neurodevelopmental outcomes and can assess neonatal brain function [15]. For these reasons, aEEG has become a research hot spot in the field of neuro-electro physiology in recent years. To better detect the neuromata development of high-risk infants, the validity of amplitude-integrated aEEG in early neuromata development of high-risk infants was investigated. Amplitude-integrated aEEG was used to monitor 100 high-risk infants. At the age of 1 year, the motor scale and validity predictors in the bailey infant and child development scale were used to assess whether or not high-risk children had neurobehavioral dysplasia. Experimental results show that amplitude-integrated aEEG can accurately and effectively predict the outcome of early neuromata development in high-risk infants. Compared with the traditional neurological examination method, it has higher sensitivity, specificity, positive predictive value, and consistency in predicting the outcome of early neuromata development in high-risk infants, which is suitable for application and promotion in China and has a better application value.

## 2. Materials and Methods

### 2.1. The Research Object

This article selects 100 cases of high-risk children with neurological developmental defects treated in paediatrics from January 2017 to December 2018 as observation objects. There were 60 males and 40 females. There were 57 high-risk infants with a gestational age less than 37 weeks. There were 35 high-risk infants with a gestational age of 37 to 40 weeks. There were 8 high-risk infants with a gestational age greater than 40 weeks. The study was approved by the hospital ethics committee, and all children's families signed informed consent. There were no significant differences in high-risk infants in terms of gender, gestational age, birth weight, 1-minute Apgar score, and delivery methods (down delivery and caesarean delivery), as shown in Table 1.

The inclusion criteria are as follows:

1. All are high-risk children, including high-risk factors such as preterm birth, intracranial haemorrhage, asphyxia, ischemic hypoxic encephalopathy, or purulent meningitis;
2. There are neurological complications, such as epilepsy, central nervous system infection, and nervous system bleeding;
3. The family members of the children were informed and signed the informed consent.

The exclusion criteria are as follows:

1. Congenital nervous system diseases or central nervous system malformations, genetic metabolic diseases, central nervous system infections, neonatal hypoglycaemia, respiratory diseases, and other serious system malformations;
2. High-risk children with abnormal chromosomes;
3. High-risk children who cannot complete aEEG as planned;
4. High-risk children who cannot be followed up till 1 year old.

### 2.2. Monitoring Method in aEEG

#### 2.2.1. Equipment

**Figure 1:**
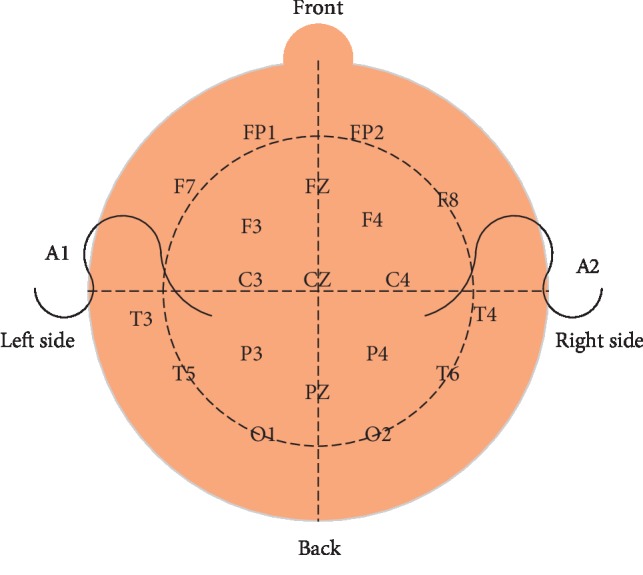
Schematic diagram of electrode placement.

The instrument used in this study is a 24-lead digital video EEG and brain function monitor. The EEG box is biologic, and the software system is XLTEK. Disposable electrodes are used. After being cleaned locally with sterile water before tracing, after preparation of the skin with scrub, Ag/AgCl disc electrodes are placed, and an appropriate amount of conductive glue is added. The breathable application fixes the skin and prevents the electrodes from moving. Turn on the power and calibrate the instrument. The electrodes are placed in the central area on both sides (C3 and C4 of the electrode positions according to the international 10–20 electrode placement system [16]). The distance between the two electrode points is 75 mm. The reference electrode is placed on the forehead midline 25 mm from the centre of the top of the head, refer to Figure 1. Record the C3-C4 original EEG signal, and process the signal through the computer's special amplification and filtering algorithm. Finally, amplitude, time compression, and rectification are performed to form the final aEEG signal. The EEG signal is output on the recording paper in a semi-logarithmic form or displayed digitally (0∼100 *μ*v), and the paper feed speed is 6 cm/h. And use artificial visual qualitative analysis.

#### 2.2.2. Qualitative Interpretation Criteria in aEEG

According to different aEEG background activity patterns [17] and sleep-wake cycle (SWC) proposed by previous studies, the classification and interpretation were performed.

**Figure 2:**
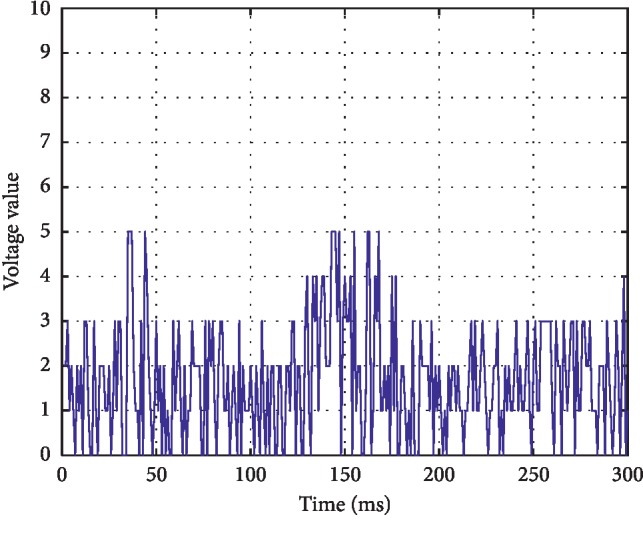
Continuous low-voltage mode below 5 *μ*v.

*(1) Flat Tracing (FT)*. Continuous low-voltage mode below 5 *μ*v, which is in the state of electrical rest, mainly electrical static. The waveform is shown in Figure 2.

**Figure 3:**
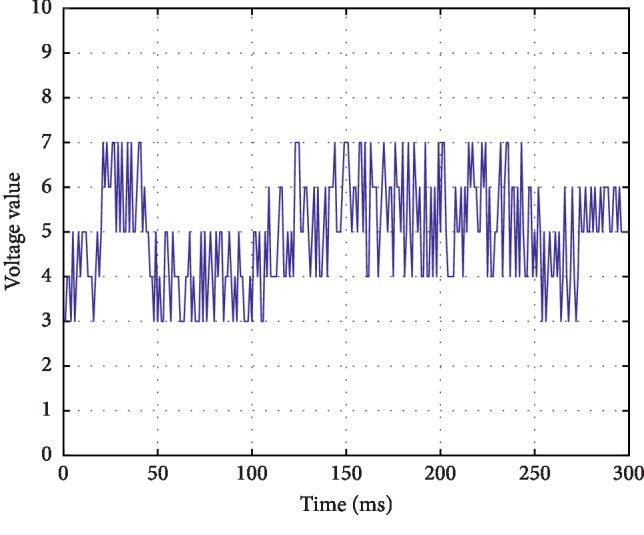
Continuous low-voltage mode at around or below 5 *μ*v.

*(2) Continuous Extremely Low Voltage (CLV)*. Continuous background low-voltage mode, mostly fluctuating around 5 *μ*v or below. The waveform is shown in Figure 3.

**Figure 4:**
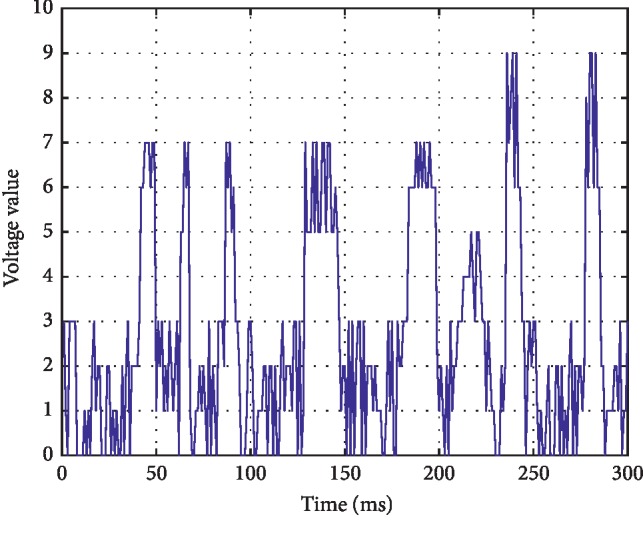
EEG patterns between extremely low voltages.

*(3) Burst-Suppression (BS)*. Discontinuous background mode, EEG mode with high amplitude bursts between extremely low voltages (electrical standstill). The waveform is shown in Figure 4.

**Figure 5:**
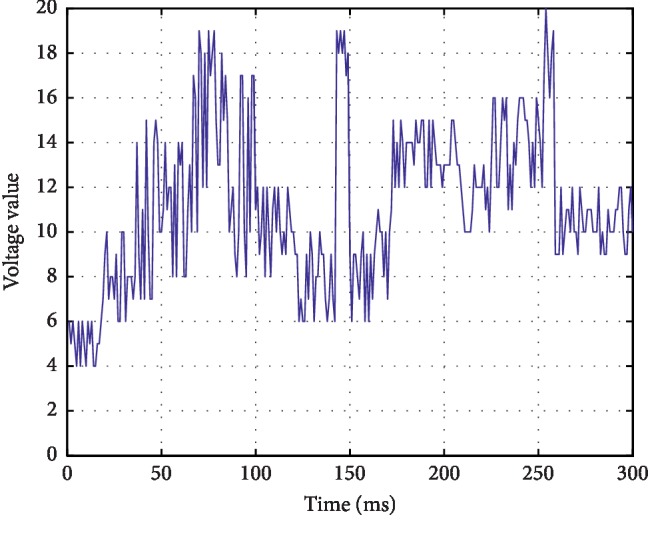
Discontinuous voltage mode above 5 *μ*v.

*(4) Discontinuous Normal Voltage (DNV)*. Discontinuous background mode, the voltage is mostly higher than 5 *μ*v. The waveform is shown in Figure 5.

**Figure 6:**
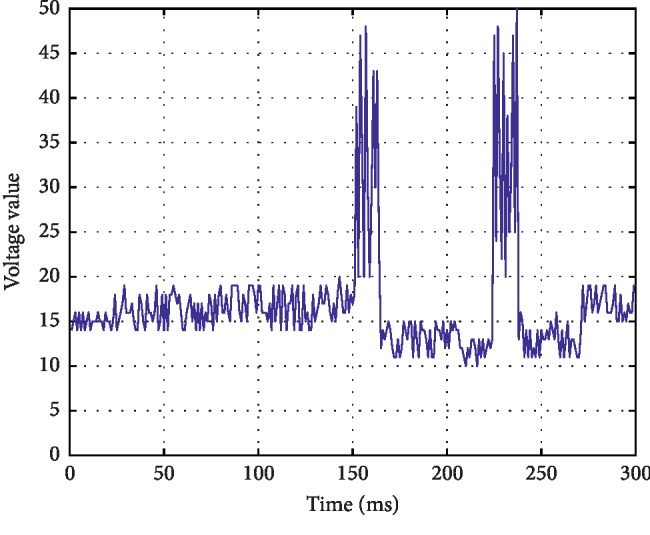
Continuous normal voltage mode.

*(5) Continuous Normal Voltage (CNV)*. Continuous EEG activity, the lower boundary of voltage is 5∼10 *μ*v and the upper boundary is 10∼50 *μ*v. The waveform is shown in Figure 6.

According to the clinical significance of aEEG, in the above five images, CNV is normal aEEG, DNV is mildly abnormal aEEG, and the rest are severely abnormal aEEG.

SWC is divided into the following three types: broadband is the sleeping period of the newborn, and narrowband is the awakening period. Mature SWCs show most of the aEEG traces analysed. Its amplitude mainly shows that the sine rhythm changes regularly (relatively stable duration and frequency), and each cycle is ≥20 minutes. If the trace pattern changes periodically, but does not meet the criteria, the mature SWC diagnostic criteria mentioned above are intermediate SWC. If no cycle-like changes occur, it is recorded as no SWC.

#### 2.2.3. Monitoring Requirements

Within 1 year after birth, all newborns were examined aEEG by 3 times. The interval between adjacent inspections is at least 3 months. When the two adjacent results are inconsistent, the second result is taken as the final result. All newborns are in a supine position in the crib and dress as little as possible to ensure that the wrist, elbow, knee, and ankle are exposed. The temperature of the room at the time of the check matches the age and clothing of the baby. Try to record when you are awake, and avoid crying, irritability, continuous snoring, or using a soother. At the age of one year, the exercise scale and predictive validity indicators in the bailey infant development scale assess the high-risk newborns for neurobehavioral abnormalities.

**Figure 7:**
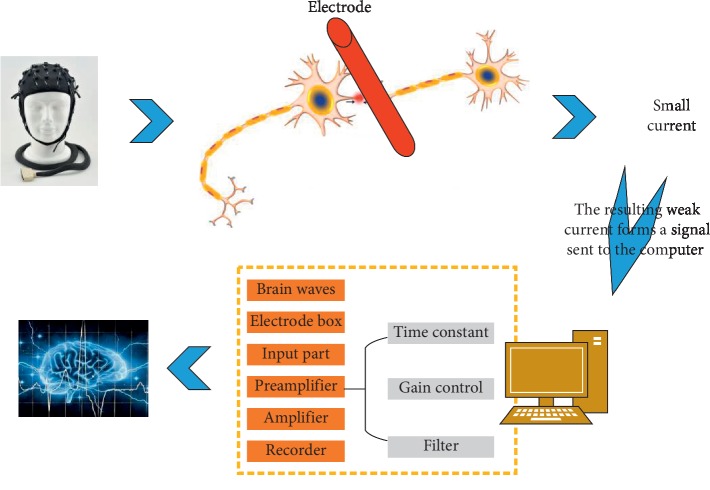
The schematic diagram of aEEG.

The schematic diagram of aEEG is shown in Figure 7. Electroencephalography is a device that provides a picture of brain activity. When connected to the head, the sensors transmit brain impulses to a machine, which maps them out in lines on an image. Normal, abnormal, or excited brain activity keeps drawing different shapes of lines on the image. Magnetoencephalography is a technique related to electroencephalography, which records electrical impulses in the brain based on changes in the magnetic field in the brain.

(1) Bailey infant development scale
High-risk children who entered the follow-up were evaluated at the age of 1 years using the exercise scale in the bailey infant development scale. The exercise scale includes coarse exercise and fine exercise, with a total of 81 items, expressed as psychomotor development index (PDI). PDI is divided into 7 levels: <69 for developmental delay; 70 to 79 for critical status; 80 to 89 for middle and lower; 90 to 109 for middle; 110 to 119 for middle and upper; 120 to 129 for excellent; and >130 points are excellent.
(2) Validity prediction indicators
  - Sensitivity: the proportion of patients who are positive with a diagnostic test;
  - Specificity: the proportion of nonill patients who are negative by diagnostic tests;
  - Positive predictive value: of the samples that are positive by the diagnostic test, the proportion of truly sick people is the positive predictive value;
  - Negative predictive value: the proportion of truly disease-free children in the samples tested negative by diagnostic tests is the negative predictive value.
(3) Statistical analysis
Data analysis was performed using SPSS 13.0 software. The two assessment methods were compared using sensitivity, specificity, positive predictive value, and negative predictive value. The consistency with the end-stage neurobiological outcome was compared using kappa value for statistical analysis, *P* < 0. 05 indicates that this method is statistically significant.

## 3. Results and Discussion

### 3.1. Disease Distribution Monitored by aEEG

**Figure 8:**
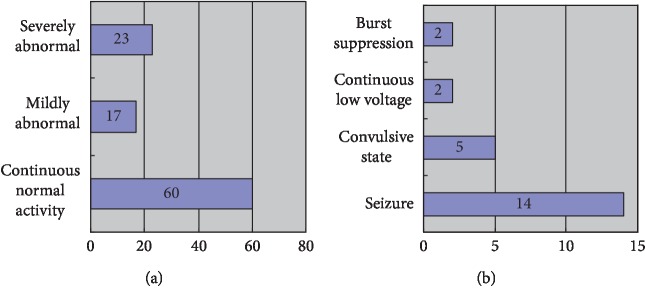
aEEG background activity pattern distribution of 100 high-risk neonates. (a) The number of cases with different background activity. (b) The number of cases in different categories of severe abnormalities.

Among the 100 subjects included, the background activity pattern of aEEG was divided into the following: 60 cases were continuous normal activities, 17 cases were mild abnormalities, and 23 cases were severe abnormalities. Of the 23 severe abnormalities, 14 were seizures, 5 were convulsions, 2 were continuous low voltage, and 2 were burst suppression. The results are shown in Figure 8.

**Figure 9:**
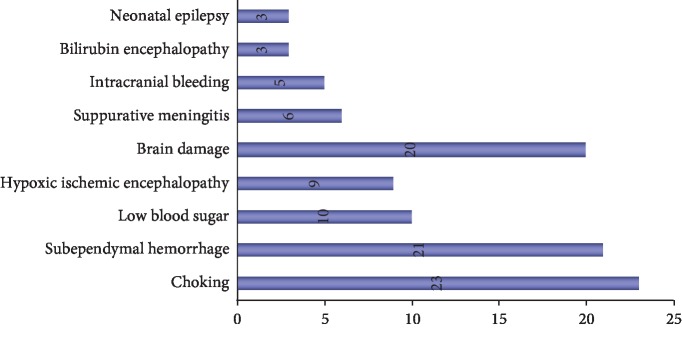
Proportion of disease distribution in 100 high-risk neonates.

A total of 100 high-risk children were included in this article. According to the nature of the case, it can be divided into 23 cases of asphyxia, 21 cases of subventricular haemorrhage, 10 cases of hypoglycaemia, 9 cases of ischemic hypoxic encephalopathy, 20 cases of brain injury, 6 cases of purulent meningitis, 5 cases of intracranial haemorrhage, 3 cases of bilirubin encephalopathy, and 3 cases of neonatal epilepsy, as shown in Figure 9.

### 3.2. Evaluation of Exercise Scale in the Bailey Infant Development Scale Monitored by aEEG

When the study subject was 1 year old, the results evaluated by the exercise scale in the bailey infant development scale were less than 69 points in 3 cases, 70 to 79 points in 8 cases, 80 to 89 points in 16 cases, and 90 to 109 in 21 cases, 110 to 119 points in 35 cases, 120 to 129 points in 12 cases, and >130 points in 5 cases, as shown in Table 2. It can be seen from Table 2 that the lower the score, the more abnormal situations occur.

### 3.3. Outcomes and Validity Prediction of Neuromotor Development Monitored by aEEG

**Figure 10:**
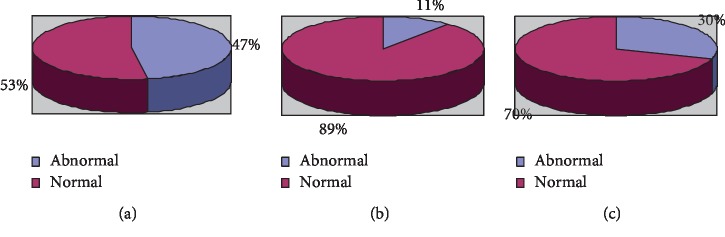
Relationship between gestational age and neuromotor development outcomes. (a) Gestational age >37. (b) 37 < gestational age <40. (c) Gestational age <40.

At 12 months of age, 67 cases had normal neuromotor development; 33 cases had delayed neuromot voltage valuator development. Neuromotor development outcomes are shown in Table 3. In early-risk infants with a gestational age less than 37 weeks, there were 27 abnormal cases of early neuromotor developmental outcomes and 30 normal cases. In high-risk infants with a gestational age between 37 and 40 weeks, there were 3 cases of abnormal neuromotor developmental outcomes. There were 3 cases of abnormal gestational age over 40 weeks. As shown in Figure 10, by plotting the gestational age of neonates and the number of neuromotor development outcomes, it can be seen that the early neuromotor development outcomes of high-risk children are related to the neonatal gestational age. Whether it is a premature infant or a newborn who has been in the mother's body for a long time, there are more abnormalities in early neuromotor developmental outcomes than in newborns born at a normal gestational age.

Table 4 shows the validity prediction results of aEEG monitoring and GMs monitoring for 100 cases. These include sensitivity, specificity, positive predictive value, and negative predictive value.

It can be seen from Table 4 that from the perspective of sensitivity, specificity, positive predictive value, and negative predictive value, the aEEG's validity prediction results are better than GMs. Therefore, the aEEG method for monitoring has better validity; it is predicted that abnormal monitoring of early neuromotor developmental outcomes in high-risk infants can be achieved.

In the neonatal period, a variety of high-risk factors can cause brain damage, which may cause a variety of neurological sequelae in the growth and development of the newborn, including cerebral palsy, mental retardation, motor retardation, and epilepsy. The early clinical manifestations of brain injury in some newborns are atypical, and early diagnosis is difficult, which often leads to missed diagnosis. When the diagnosis is clear, the best period of treatment and intervention has been missed. Therefore, it is important to determine early brain damage and accurately predict neurodevelopmental outcomes in high-risk neonates.

The results of this study show that aEEG can predict the sensitivity, specificity, positive predictive value, and negative predictive value of high-risk neonatal adverse neurodevelopmental outcomes, which are 90.631%, 94.125%, 87.887%, and 95.529%, respectively. The results are quite higher than those predicted by the GMs quality assessment. This shows that aEEG surveillance has a better predictive value for adverse neuromata development outcomes in high-risk neonates.

## 4. Conclusions

In recent years, with the gradual improvement of perinatal health care and the advancement of neonatal intensive care and rescue technology, the survival rate of perinatal high-risk neonates has increased significantly convulsions, meningitis, respiratory failure, hyperbilirubinemia, and other newborns, even after rescue, whether there are abnormal neurobiological outcomes is worrying. Inspections are becoming increasingly important. In this article, through the study of 100 high-risk children, and by predicting the validity of aEEG monitoring in early neuromotor development outcomes in high-risk children, it is confirmed that there is a correlation between aEEG monitoring and neuromotor development outcomes. When aEEG monitoring is abnormal in high-risk neonates, it is highly suspected that the child's neuromotor developmental outcome is poor, and treatment measures need to be taken as soon as possible. Early intervention reduces the continued damage of the children's brain and nerves and improve the sequelae. The shortcoming of this article is that the follow-up time of the child is short, only corrected to 1 year, and the child needs to be followed up for a longer time to better understand the relationship between aEEG monitoring and neuromotor development outcomes, and to understand the aEEG in predictive value of monitoring. Our next step is to carry out a longer follow-up to better understand the relationship between aEEG monitoring and neuromata developmental outcomes, and to better understand the predictive value of aEEG monitoring.

## Figures and Tables

**Table 1:** Results of general clinical data for high-risk children.

Group	Number of cases	Male/female	Gestational age	Birth weight (g)	1 min Apgar (minutes)	Method of delivery
High-risk child	100	51/49	32.2 ± 4.5	1912.9 ± 0.2	8.9 ± 1.01	30/38
*χ* ^2^/*t*		1.412	1.281	1.512	1.201	1.541
*P*		0.121	0.135	0.101	0.129	0.125

**Table 2:** Results of 52 neuromotor examination scores and neuromotor development outcomes from 0 to 1 year old.

Score	Number	Torsional phase		Restless movement	
		Abnormal	Normal	Abnormal	Normal
Score <69	3	3	0	3	0
70 <score <79	8	7	1	8	0
80 <score <89	16	11	5	10	5
90 <score <109	21	9	12	10	11
110 <score <119	35	2	33	3	32
120 <score <129	12	1	11	0	12
130 <score	5	0	5	0	5

**Table 3:** Results of neuromotor development outcomes.

	Neuromotor developmental outcomes		Total
	Abnormal	Normal	
Gestational age <37 weeks	27	30	57
37 weeks < gestational age <40 weeks	3	24	35
Gestational age >40 weeks	3	7	8
Total	33	67	100

**Table 4:** Results of validity prediction of aEEG and GMs monitoring.

Inspection method	aEEG	GMs
Sensitivity	90.631%	78.381%
Specificity	94.125%	80.187%
Positive predictive degree	87.887%	65.791%
Negative predictive degree	95.529%	88.712%

## Data Availability

No data were used to support this study.
